# Isolation of Fibroblast-Activation Protein-Specific Cancer-Associated Fibroblasts

**DOI:** 10.1155/2017/4825108

**Published:** 2017-08-14

**Authors:** Yingying Huang, Sufang Zhou, Yong Huang, Duo Zheng, Qiqi Mao, Jian He, Yiwei Wang, Dabing Xue, Xiaoling Lu, Nuo Yang, Yongxiang Zhao

**Affiliations:** ^1^National Center for International Research of Biological Targeting Diagnosis and Therapy, Guangxi Key Laboratory of Biological Targeting Diagnosis and Therapy Research, Collaborative Innovation Center for Targeting Tumor Diagnosis and Therapy, Guangxi Medical University, Nanning, Guangxi, China; ^2^Shenzhen Key Laboratory of Translational Medicine of Tumor, Department of Basic Medicine, School of Medicine, Shenzhen, Guangdong 518000, China

## Abstract

The current study is to develop a gentle and efficient method for purification of fibroblast-activation protein positive (FAP^+^) cancer-associated fibroblasts (CAFs) from tumor tissues. Fresh tissues were isolated from BALB/c-Nude mice bearing human liver cancer cell line (HepG2), fully minced and separated into three parts, and digested with trypsin digestion and then treated with collagenase type IV once, twice, or thrice, respectively. Finally, the cells were purified by using FAP magnetic beads. The isolated CAFs were grown in culture medium and detected for the surface expression of fibroblast-activation protein (FAP). The number of adherent cells which were obtained by digestion process with twice collagenase type IV digestion was (5.99 ± 0.18) × 10^4^, much more than that with the only once collagenase type IV digestion (2.58 ± 0.41) × 10^4^ (*P* < 0.0001) and similar to thrice collagenase type IV digestion. The percentage of FAP^+^ CAFs with twice collagenase type IV digestion (38.5%) was higher than that with the only once collagenase type IV digestion (20.0%) and little higher than thrice collagenase type IV digestion (37.5%). The FAP expression of CAFs was quite different from normal fibroblasts (NFs). The fibroblasts isolated by the innovation are with high purity and being in wonderful condition and display the features of CAFs.

## 1. Introduction

Cancer-associated fibroblasts (CAFs) play an essential role in promoting tumor occurrence and growth [1–4]. Tumor proliferation, angiogenesis, invasion, and metastasis are dependent on the cancer-associated fibroblasts secretion of various cytokines, chemokines, growth factors, and degradation of extracellular matrix (ECM) proteins for platform [5–8]. Based on previous studies involving tumor stromal fibroblasts or extracellular matrix, the cancer-associated fibroblasts (CAFs) and normal fibroblasts (NFs) differ in biological characteristics and gene expression profile [9, 10]. When compared to normal fibroblasts, cancer-associated fibroblasts are spindle- and stellate-liked cells, with abundant cytoplasm and poor cell polarity, growth dense, and uncontrolled, highly and specifically expressed *α*-smooth muscle actin (*α*-SMA) [11]. Cancer-associated fibroblasts are responsible for the generating of cytokines and growth factors which can affect tumor cells growth and metastasis and the production and reconstruction of most extracellular matrix in tumor stroma [12]. The study of the mechanism of the development of cancer-associated fibroblasts plays a major role for tumor diagnosis and therapy. Although cancer-associated fibroblasts are the major components of cancer stroma, they are densely arranged and surrounded by connective tissue and various other cell types, embedded in matrix components [13]. Therefore, it is beset with difficulties to isolate cancer-associated fibroblasts from tumor tissue.

For the past few years, purification technology of CAFs for culture and molecular profiling has obtained more and more attention and become more and more diversified, such as using Fluorescence-Activated Cell Sorting (FACS) to isolate CAFs and NFs from fresh tissues; however, FACS is too expensive to popularize and may lose some cells during the process [14–16]. Besides, some researchers found that not all CAFs at all times express specific marker fibroblast-activation protein (FAP) [17]. These isolated techniques all tend to got only a small number of wanted cells. This paper reports a new purification method to gain numerous FAP^+^ CAFs in good conditions.

## 2. Material and Methods

### 2.1. Cells and Animals

BALB/c-Nude mice were purchased from Vital River Company (Beijing, China) and were housed and cared in accordance with the Federation of European Laboratory Animal Science Association guidelines, and all protocols were approved by the Animal Ethics Committee of Guangxi Medical University (Nanning, Guangxi, China). Human liver carcinoma cells (HepG2) (1 × 10^6^) were injected into the left flank of mice. 50 days after injection, tumors were excised for study.

### 2.2. Cancer-Associated Fibroblasts (CAFs) Isolation (Figure 1)

Tumors were separated and placed in 37°C PBS solution for 1–3 min. Peripheral and necrotic tissues were removed and remaining tumor was fully cut up by using an ophthalmic scissor. Dissociation of 0.2 × 0.2 × 0.2 cm^3^ minced tissues was digested in 5 ml trypsin solution (DDH_2_O, 40 mg NaCl, 2.5 mg NaHCO_3_, 5 mg glucose, 1.5 mg ethylenediaminetetraacetic acid (EDTA), and 1.25 mg trypsin) at room temperature for 5 min and then ended by 10 ml FBS. These suspended tissues were washed by PBS solution and then were resuspended in an enzyme cocktail of 20 ml Dulbecco's modified Eagle's medium (DMEM), 2 ml FBS, and 10 mg collagenase type IV at room temperature for 5, 10, 20, 30, 45, 60, and 75 min of constant mixing with a vortex. Tissues were gently pipetted by using a pasteur pipette, PBS solution washed, and then were resuspended in 10 mL DMEM medium (containing 10% FBS), plated into 25 cm^2^ cell culture flasks and cultured in incubator (37°C, 5% CO_2_) for two days. During this time, through the microscope, some adherent cells were found climbing out from these suspension loose tumor tissues. The rest of the suspension loose tumor tissues was through the above digestion process for the second time and the third time. Single cells were magnetically labeled with anti-FAP microbeads (Miltenyi Biotec, Bergisch Gladbach, Germany) in the dark at 4°C for 30 min and applied to the prepared MS Column (Miltenyi Biotec, Bergisch Gladbach). FAP^−^ cells were collected in the flow-through of the column, while FAP^+^ cells bound to the beads were flushed out by applying the plunger supplied with the column. Sorted FAP^+^ cells were plated into six-well plates and cultured in DMEM medium (containing 10% FBS, ScienCell, USA).

### 2.3. Immunocytochemistry/Immunofluorescence (ICC/IF)

Adherent cells that have been isolated were plated into six-well plates at 1 × 10^5^ cells/dish. At 70% confluence, serum-free DMEM was used as culture medium for 24 h. Cells were washed and fixed with 4% paraformaldehyde and then incubated in 10% normal serum from the secondary antibody species at room temperature for 30 min to block nonspecific protein-protein interactions. Cells were then incubated with primary antibody FAP or *α*-SMA (1 *μ*g/ml) (Abcam, Cambridge, UK) at 4°C overnight. Then this was followed by Alexa Fluor 594-conjugated secondary (1/400) or Alexa Fluor 488-conjugated secondary (1/400) (Abcam, Cambridge, UK) at room temperature for 30 min and DAPI staining for 3 min. The antigen-antibody binding was assessed with fluorescence microscopy.

### 2.4. Fluorescence-Activated Cell Sorting (FACS)

For flow cytometry, cells were stained at the concentration of 1 × 10^6^ cells/90 *μ*l buffer and 10 *μ*l phycoerythrin-conjugated antibody FAP (R&D Systems, Minneapolis, US) at 4°C for 30 min before FACS analysis. All data were analyzed by EXPO32 Software.

### 2.5. Western Blotting

Cells were washed at 4°C and lysed in RIPA buffer on ice. Then, cell lysates were centrifuged at 13,000*g* at 4°C for 20 min. Total protein concentration in the cell lysate was measured by BCA Protein Quantification kit (Beyotime Biotechnology). Cell lysates were separated by SDS-PAGE and transferred onto a nitrocellulose membrane. The nitrocellulose membrane was incubated with primary anti-FAP antibody at 4°C overnight. After washing the nitrocellulose membrane with TBS-T three times for 10 min each at room temperature, the nitrocellulose membrane was incubated with horseradish peroxidase- (HRP-) conjugated anti-IgG Abs (ZSGB-Bio, Beijing, China). Protein band intensities were quantified by use of the diaminobenzidine (DAB) kit (SolarBio, Beijing, China). *β*-Actin was used as internal control.

### 2.6. Statistical Analysis

Data are showed as mean ± SEM. The significance of differences between groups was assessed by *t*-test. All analyses were performed with GraphPad Prism program version 5 (GraphPad Software, California, USA).

## 3. Results

### 3.1. Isolation of CAFs

**Figure 2:**
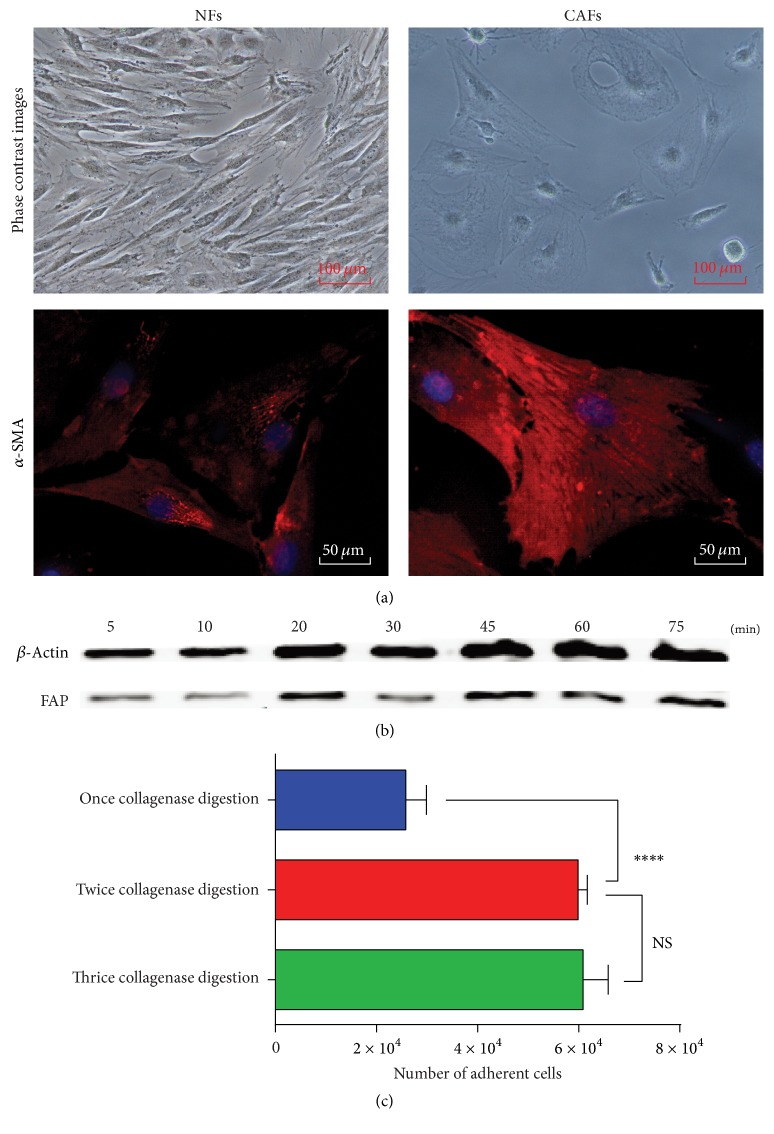
Cancer-associated fibroblasts (CAFs) colonies from tumor before purification by magnetic beads. (a) Phase contrast images and *α*-SMA expression of CAFs after isolation by different digestion procedures. Normal fibroblasts (NFs) as negative control cells isolated from normal human tissue (such as skin) (scale bars represent 100 *μ*m and 50 *μ*m). (b) Western blot analysis showed the FAP expression of adherent cells by different time duration of collagenase type IV digested with a vortex. (c) The statistical chart of the number of adherent cells after isolation by different digestion procedures. Data are presented as the mean ± SEM (*n* = 3). NS, not significant; ^*∗∗∗∗*^*P* < 0.0001.

Normal fibroblasts (NFs) isolated from normal human skin were small spindle cells, while CAFs colonies from tumor were stelliform cells with higher *α*-SMA expression (Figure 2(a)). FAP^+^ cells reached highest level when collagenase type IV digested for 45 min with a vortex (Figure 2(b)). Before magnetic separation, the number of adherent cells digested with twice collagenase type IV digestion ((5.99 ± 0.18) × 10^4^) was much higher than the one with only once collagenase type IV digestion ((2.58 ± 0.41) × 10^4^, *P* < 0.0001) similar to the number of thrice collagenase type IV digestion ((6.09 ± 0.50) × 10^4^, NS, not significant) (Figure 2(c)).

### 3.2. Enrichment and Purity of FAP^+^ CAFs

**Figure 3:**
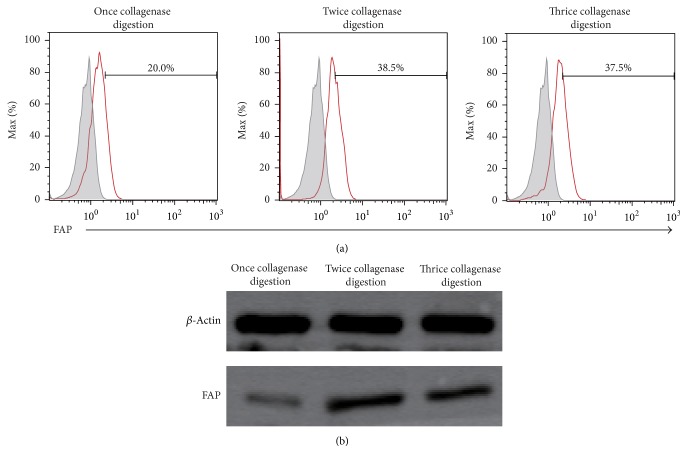
FAP expression of CAFs before purification by magnetic beads. (a) Fluorescence-Activated Cell Sorting (FACS) showed the FAP expression of CAFs. (b) Western blot analysis showed the FAP expression of CAFs.

**Figure 4:**
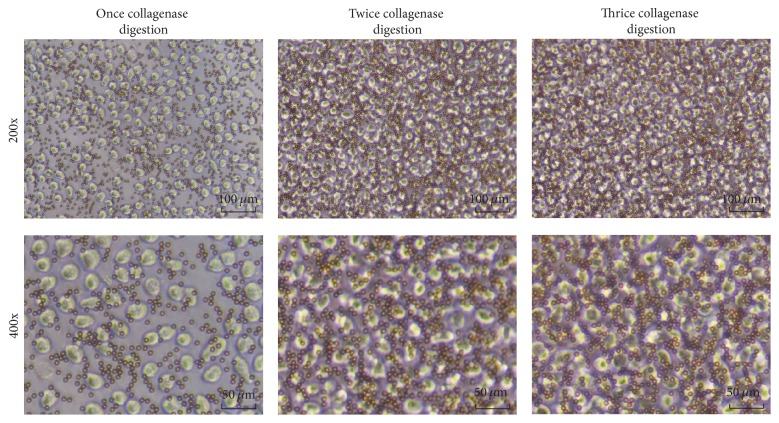
CAFs combined with magnetic beads (scale bars represent 100 *μ*m and 50 *μ*m).

Before purification by magnetic beads, the percentage of FAP^**+**^ CAFs digested with twice collagenase type IV digestion (38.5%) was much higher than that with only once collagenase type IV digestion (20.0%) and little higher than the number of thrice collagenase type IV digestion (37.5%) (Figure 3). After magnetic cell separation (MACS), we showed that FAP^**+**^ CAFs are combined with microbeads as detected under a microscope (Figure 4).

### 3.3. Detection of FAP^+^ CAFs Activity

**Figure 5:**
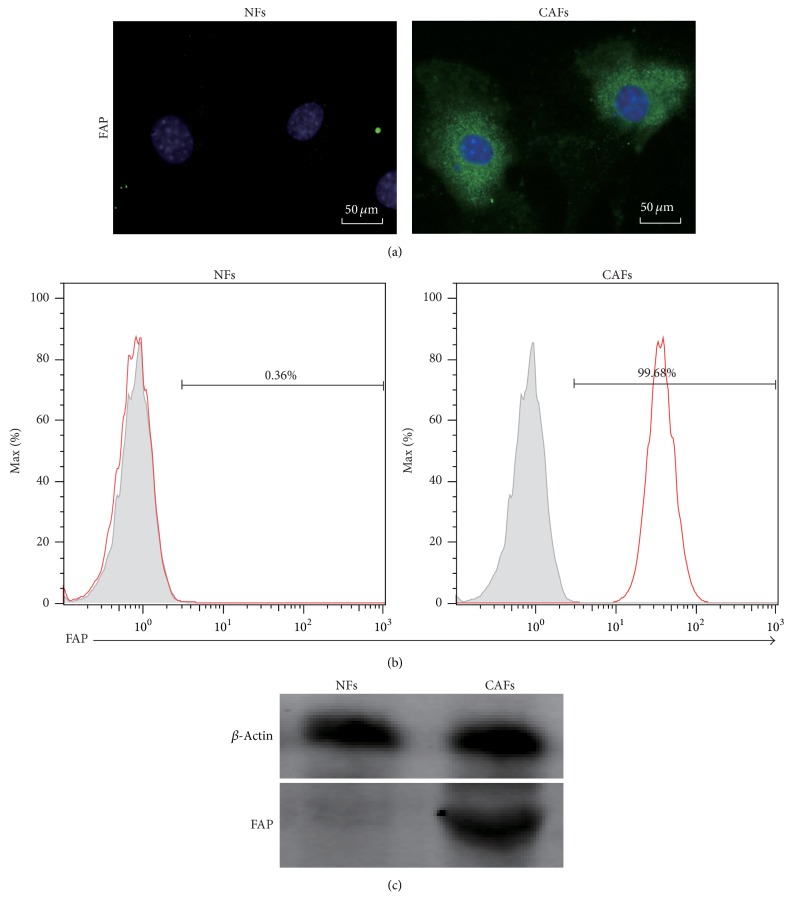
FAP expression of CAFs after purification by magnetic beads. (a) Immunocytochemistry/immunofluorescence (ICC/IF) results of FAP^+^ CAFs (scale bars represent 50 *μ*m). (b) Fluorescence-Activated Cell Sorting (FACS) showed the FAP expression of CAFs. (c) Western blot analysis showed the FAP expression of CAFs. Normal fibroblasts (NFs) as negative control cells isolated from normal human tissue (such as skin).

Immunocytochemistry/immunofluorescence (ICC/IF) showed that FAP^**+**^ CAFs after digestion with twice collagenase type IV digestion and purification by magnetic beads exhibited strong fluorescence in cytoplasm (Figure 5(a)). The purity of the FAP^**+**^ CAFs was 99.68%, while NFs which were isolated from normal human skin without FAP expression (Figures 5(b) and 5(c)).

## 4. Discussion

In the process of the tumor occurrence and growth, tumor-associated fibroblasts are emerged as essential inducement [18]. Moreover, the tumor microenvironment created by tumor-associated fibroblasts is always dominant during the process of tumor development. It is well known that, during tumor development, tumor-associated fibroblasts not only undergo cellular and molecular changes but also affect other normal stromal cells transformation, which accompany the appearance of tumor malignancy and invasion [19–21].

Cancer-associated fibroblasts, a major component of the cancer stroma, are necessary for tumor growth and metastasis [22, 23]. FAP, *α*-SMA, FSP1, and PDGFR are not only markers that have important role in cancer-associated fibroblasts but also essential for the proliferation of tumor cells and stimulating metastasis of tumors [17, 24].

In view of the known heterogeneity of tumor-associated fibroblasts, it would appear reasonable to study tumor-associated fibroblasts isolated from tumor tissues when studying the mechanisms of tumor progression [25–27]. Even though methods have been reported for the isolation of cancer-associated fibroblasts, the purity and efficiency of sorting have not been described.

In the present work, we reported a method to isolate FAP^+^ CAFs, an important cell population found in tumor tissue, through twice collagenase type IV digestion and magnetic cell separation. This purification technique produces isolated cells with purity in excess of 99.68%, and much more number and better condition of FAP^+^ CAFs than that with once and thrice collagenase type IV digestion. The specific markers of cancer-associated fibroblasts (CAFs) are diametrically opposite to normal fibroblasts (NFs). This technology counts a great deal role in carcinogenesis.

## Figures and Tables

**Figure 1:**
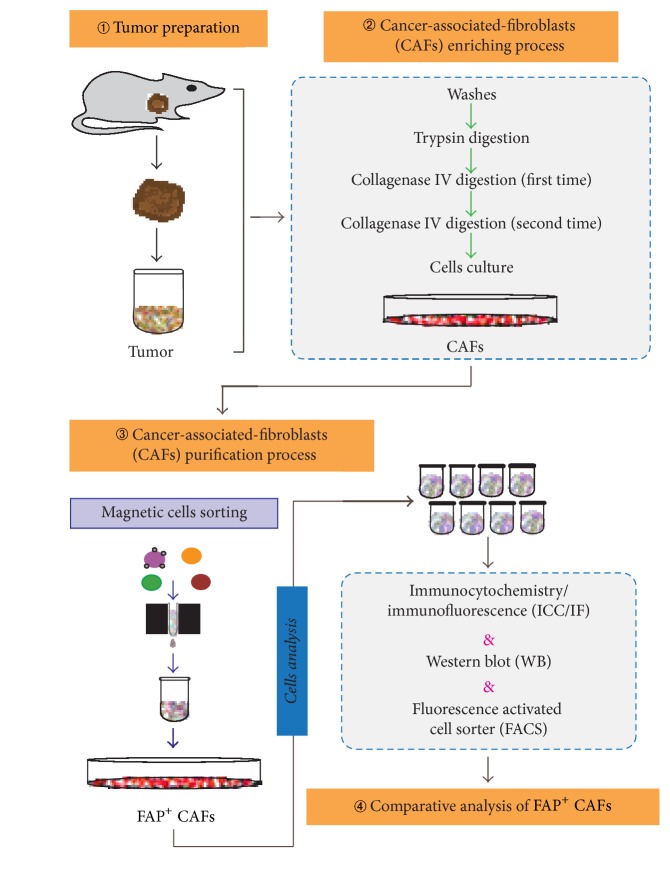
Flow chart and overview of the FAP^+^ CAFs isolation procedure.
